# Maintaining High Strength in Mg-LPSO Alloys with Low Yttrium Content Using Severe Plastic Deformation

**DOI:** 10.3390/ma11050733

**Published:** 2018-05-05

**Authors:** Gerardo Garces, Sandra Cabeza, Rafael Barea, Pablo Pérez, Paloma Adeva

**Affiliations:** 1 Departamento de Metalurgia Física, Centro Nacional de Investigaciones Metalúrgicas, CENIM, CSIC, Avda. Gregorio del Amo 8, 28040 Madrid, Spain; zubiaur@cenim.csic.es (P.P.); adeva@cenim.csic.es (P.A.); 2 Institute Laue-Langevin, ILL, 38042 Grenoble, France; cabeza@ill.fr; 3 Departamento de Ingeniería Industrial, Universidad Nebrija, Campus Dehesa de la Villa, C. Pirineos 55, 28040 Madrid, Spain; rbarea@nebrija.es

**Keywords:** magnesium alloys, ECAP, powder metallurgy, mechanical properties, LPSO-phase

## Abstract

Alternative processing routes such as powder metallurgy, the extrusion of recycled chips, or equal channel angular pressing (ECAP) have been considered for effective methods of maintaining the high mechanical strength of Mg-Y-Zn alloys containing long-period stacking ordered structures with respect to the alloy processed by the conventional extrusion of as-cast ingots with the advantage of minimizing the yttrium content. A yield stress similar to that found for extruded Mg_97_Y_2_Zn_1_ alloy can be attained with only half of the usual yttrium and zinc additions thanks to the grain refinement induced by ECAP processing. The properties of Mg_98.5_Y_1_Zn_0.5_ subjected to ECAP are maintained up to 200 °C, but superplastic behavior is found above this temperature when the alloy is processed through a powder metallurgy route.

## 1. Introduction

The mechanical strength and creep resistance of Mg-Y,RE-Zn (RE = rare earths) alloys containing long-period stacking ordered (LPSO) phases at intermediate temperatures exceed by far those of commercial magnesium alloys [1,2,3,4,5,6]. LPSO-phases are solid solutions of yttrium or certain rare earth elements and transition metals in the magnesium lattice, where these atoms are arranged periodically in the magnesium basal planes to form ordered structures [7,8,9,10,11]. Depending on the processing route, important variations in mechanical properties can be obtained. The Mg_97_Y_2_Zn_1_ (at %) alloy produced by the warm extrusion of rapidly solidified (RS) powders exhibited the highest high yield strength reported in this system, about 610 MPa [1]. The extrusion of RS ribbons with a very fine dendritic microstructure renders a yield stress of 410 MPa [12]. The optimization of extrusion parameters of as-cast Mg_97_Y_2_Zn_1_ ingots results in a material combining cast high-yield stress (around 350 MPa) and appreciable ductility [3]. Furthermore, subsequent equal channel angular processing (ECAP) after extrusion leads to yield stresses higher than 400 MPa due to grain refinement of the magnesium phase [13,14]. However, in all cases, the yttrium and/or RE contents were at least 2 at %. Reduction in the use of rare earth elements is a critical point in the design of new high-strength magnesium alloys because there is a tendency to minimize the content of rare earth elements since the market is controlled by China and future availability is not guaranteed. Consequently, Europe, Japan, and the USA are proposing policies/guidelines addressed to substitute the use of such elements as much as possible [15,16]. In the case of LPSO-phase containing alloys, the decrease in the Y concentration implies a significant decrease in mechanical strength because the volume fraction of the reinforcing LPSO-phase is reduced [17]. Therefore, an additional refinement of the grain size in alloys containing the LPSO-phase is needed in such a way that it can compensate minor hardening due to the smaller volume fraction of the LPSO-phase. At this point, the use of a powder metallurgy route or severe plastic deformation (SPD) techniques are proposed in the present study as suitable methods for refining the grain size of the alloy.

An alloy with a composition of Mg_98.5_Y_1_Zn_0.5_ (at %) (with half the yttrium concentration of Mg_97_Y_2_Zn_1_ alloy) was processed by three different techniques: ECAP, the consolidation of machining chips (CM), and powder metallurgy (PM). Therefore, the present study examines the microstructure and the mechanical strength from room temperature up to 300 °C of the Mg_98.5_Y_1_Zn_0.5_ alloy processed through these alternative processes. Results are compared to those obtained in Mg_97_Y_2_Zn_1_ alloy processed by the extrusion of the as-cast material.

## 2. Materials and Methods 

The alloy Mg_98.5_Y_1_Zn_0.5_ (at %) was obtained by melting high purity Mg and Zn as well as a Mg-22%Y (wt %) master alloy in an electric resistance furnace. The mixture was then cast in cylindrical steel molds of 42 mm in diameter. Machined chips and rapidly solidified (RS) powders were obtained from the cast bars. On one hand, the machined chips were fabricated by high-speed machining in a conventional Computer numerical controlled (CNC) lathe. RS powders were prepared by the EIGA (Electrode Induction Melting Gas Atomization) process by TLS Technik GmbH (Bitterfeld-Wolfen, Germany). Powders have a spherical morphology with a diameter less than 100 µm. Chips and powders were uniaxial compacted under 350 MPa for 2 min at room temperature (RT), providing cylindrical green compacts of 40 mm in diameter. Chip and powder compacts and cast cylinders machined up 40 mm were extruded at 350 °C using several extrusion ratios depending on the material: 4:1 and 18:1 for the cast alloy and 36:1 for the chip and powder compacts. A higher extrusion ratio is required for chips and powders to ensure their complete consolidation.

**Figure 1:**
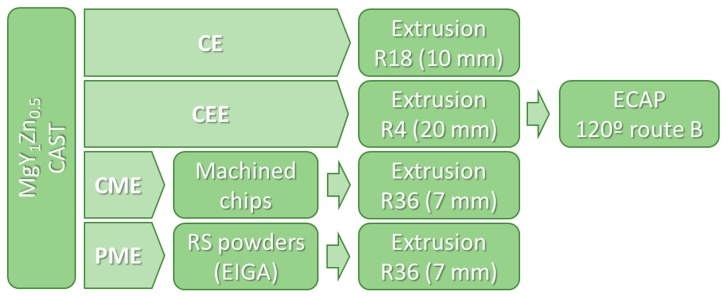
Schematic view of the production methods for the Mg_98.5_Y_1_Zn_0.5_ alloy: CE, CME, CEE and PME.

Cylinders from the extruded cast alloy bar, 70 mm long and 20 mm in diameter (extrusion ratio 4:1) were ECAP processed at 300 °C, using route B (i.e., 90° rotation of the sample between passes). A hydraulic machine with a circular cross-section die with a diameter of 20 mm and a die angle of 118° was used. Per pass, a true strain of 0.7 was produced. For ECAP process, samples were heated in the die, reaching die temperature in 5 min before pressing. A standard pressing speed of 20 mm/min was used in all cases. Following each ECAP pass, the heated split-die was opened hydraulically for rapid sample removal and then water quenched. The alloy able to be processed for up to four passes without cracking. Figure 1 shows the schematic view of the production methods for the Mg_98.5_Y_1_Zn_0.5_ alloy. 

Microstructural characterization was carried out using scanning electron microscopy (SEM) and X-ray diffraction (XRD). The microscope JEOL JSM 6500F (JEOL, Akishima, Tokio, Japan) was used in the backscattered mode. Metallographic preparation consisted of mechanical polishing and etching in a solution of 0.5 g picric acid, 5 mL acetic acid, 20 mL ethanol, 1 mL water, and 25 mL methanol. Quantitative image analysis was carried out to follow the evolution of the recrystallized fraction and grain size in the magnesium matrix after extrusion or ECAP. For the recrystallized fraction, several SEM images from areas recrystallized to different extents were measured to give a good statistical measure of this fraction. The sizes of recrystallized grains were measured, counting a minimum of 500 grains from backscattered electrons images. Statistical analyses were carried out with the software Sigma Scan Pro (Jandel Scientific, San Rafael, CA, USA), taking the grain size as the average value obtained. XRD patterns were carried out in a Siemens diffractometer D5000 using Cu-Kα radiation with a wavelength of 0.1506 nm.

Cylindrical samples machined with their long direction parallel to the extrusion/ECAP direction (head diameter of 6 mm, curvature radius of 3 mm, gauge diameter of 3 mm, and gauge length of 10 mm) were deformed in tension at a constant strain rate of 10^−4^ s^−1^ from room temperature up to 300 °C.

For simplicity, the different processing routes were designed as follows: CE for the alloy extruded from the cast bars, CEE for the ECAP processing, CME for the alloy produced using machined chips, and, finally, PME for the alloy fabricated using RS powders.

## 3. Results and Discussion

**Figure 2:**
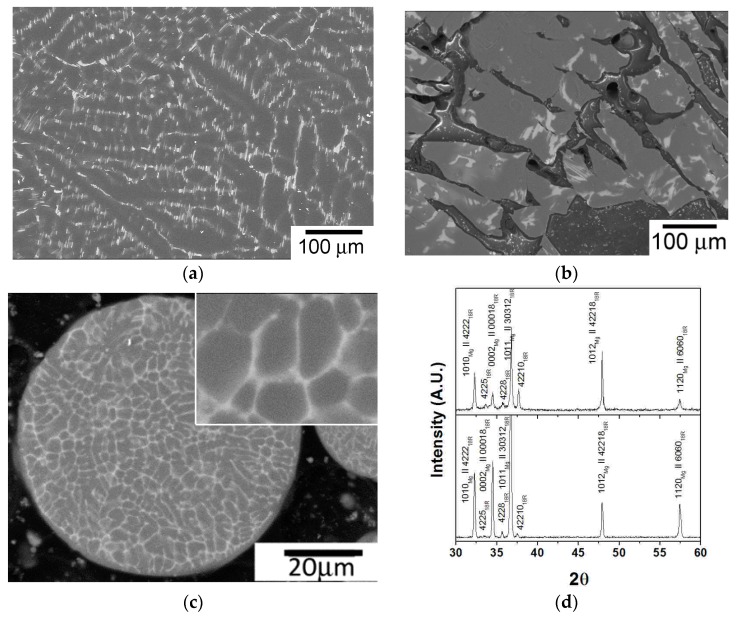
Microstructure of the Mg_98.5_Y_1_Zn_0.5_ alloy: (**a**) as-cast alloy, (**b**) machined chips, and (**c**) rapidly solidified (RS) powders. The grey and white phases correspond to the magnesium and LPSO-phases. (**d**) Diffraction pattern of the Mg_98.5_Y_1_Zn_0.5_ alloy: as-cast alloy and RS powders.

The micrograph of the Mg_98.5_Y_1_Zn_0.5_ cast alloy (Figure 2a) revealed a two-phase dendritic microstructure consisting of magnesium dendrites with a second phase located at the interdendritic space. The interdendritic phase was characterized by a lamellar morphology typical of the LPSO-phase. The volume fraction of the LPSO-phase was 9%—much lower than volume fraction in the Mg_97_Y_2_Zn_1_ alloy, which was between 20% and 25% [17,18]. The microstructure of the machined chips (Figure 2b) was similar to that of the cast alloy, but the LPSO-phase was broken. The dendritic microstructure of spherical RS powders was also dendritic, but much finer than that found in the as-cast ingots, as shown in Figure 2c. XRD patterns of Figure 2d confirmed that magnesium and 18R-LPSO-phase are the only phases that existed in the as-cast alloy and RS powders.

**Figure 3:**
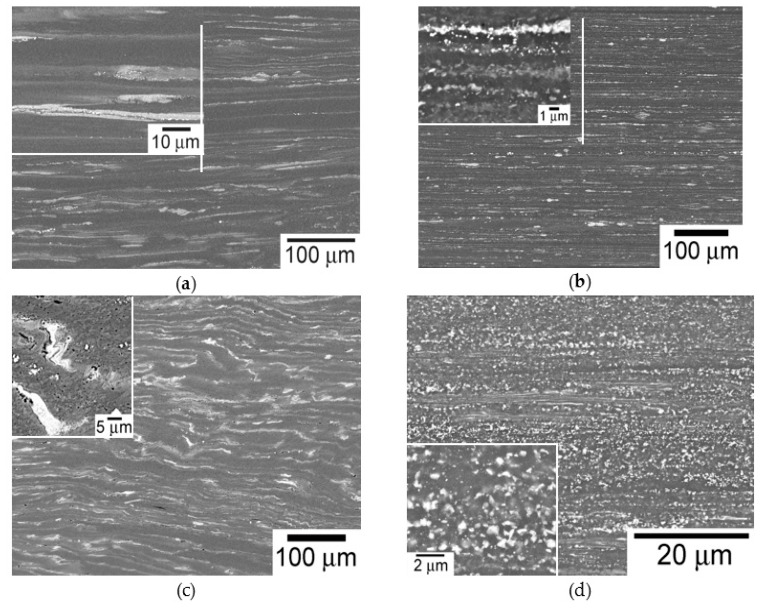
Microstructure of processed Mg_98.5_Y_1_Zn_0.5_ alloy: (**a**) CE, (**b**) CME, (**c**) CEE, and (**d**) PME. The grey and white phases correspond to the magnesium and LPSO-phases.

After extrusion, the LPSO-phase in the CE material appeared strongly elongated along the extrusion direction, independent of the extrusion ratio (Figure 3a). The LPSO-phase in the extruded bar produced using machined chips (the CME material) was also deformed and elongated along the extrusion direction (Figure 3b). However, at higher magnifications, it was observed that the LPSO-phase was fragmented into small particles with a diameter lower than 1 µm. The LPSO-phase in the CEE material was still elongated along the initial extrusion direction but also showed a serrated shape distribution after each pass by ECAP, as shown Figure 3c. During the extrusion process of the RS powders (PME material), the LPSO-phase appeared to be homogeneously and finely distributed in the magnesium matrix with a particle size below 500 nm (Figure 3d). These LPSO-phase particles were located mainly at grain boundaries, inhibiting the grain growth during the extrusion process.

**Figure 4:**
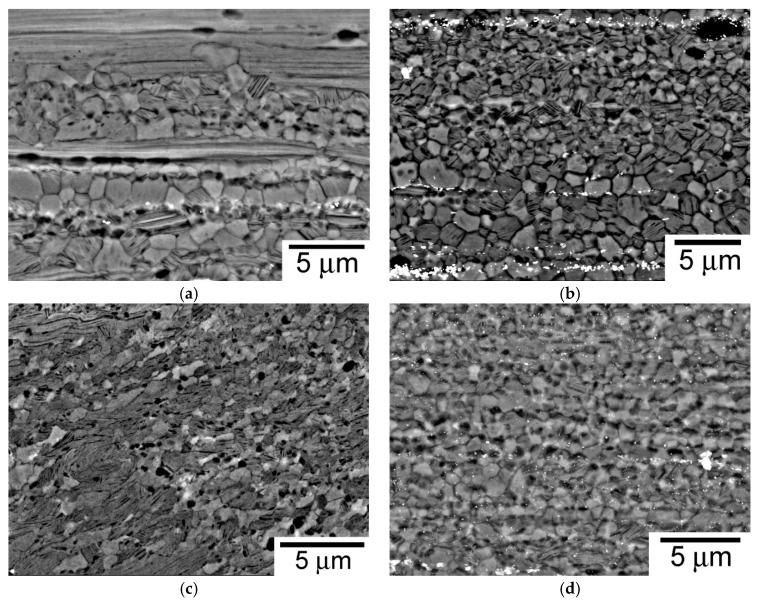
Grain structure of processed Mg_98.5_Y_1_Zn_0.5_ alloy: (**a**) CE; (**b**) CME; (**c**) CEE; and (**d**) PME.

The grain structure of the alloy processed by the four routes is shown in Figure 4. The grain structure depends on the processing route; it was (i) equiaxed for PME and CME alloys, and (ii) bimodal, in which fine dynamically recrystallized (DRXed) and coarse non-recrystallized grains coexist. Using image analysis techniques, the ratio of DRXed and non-DRXed regions as well as the size of DRXed grains were evaluated (see Table 1).

**Figure 5:**
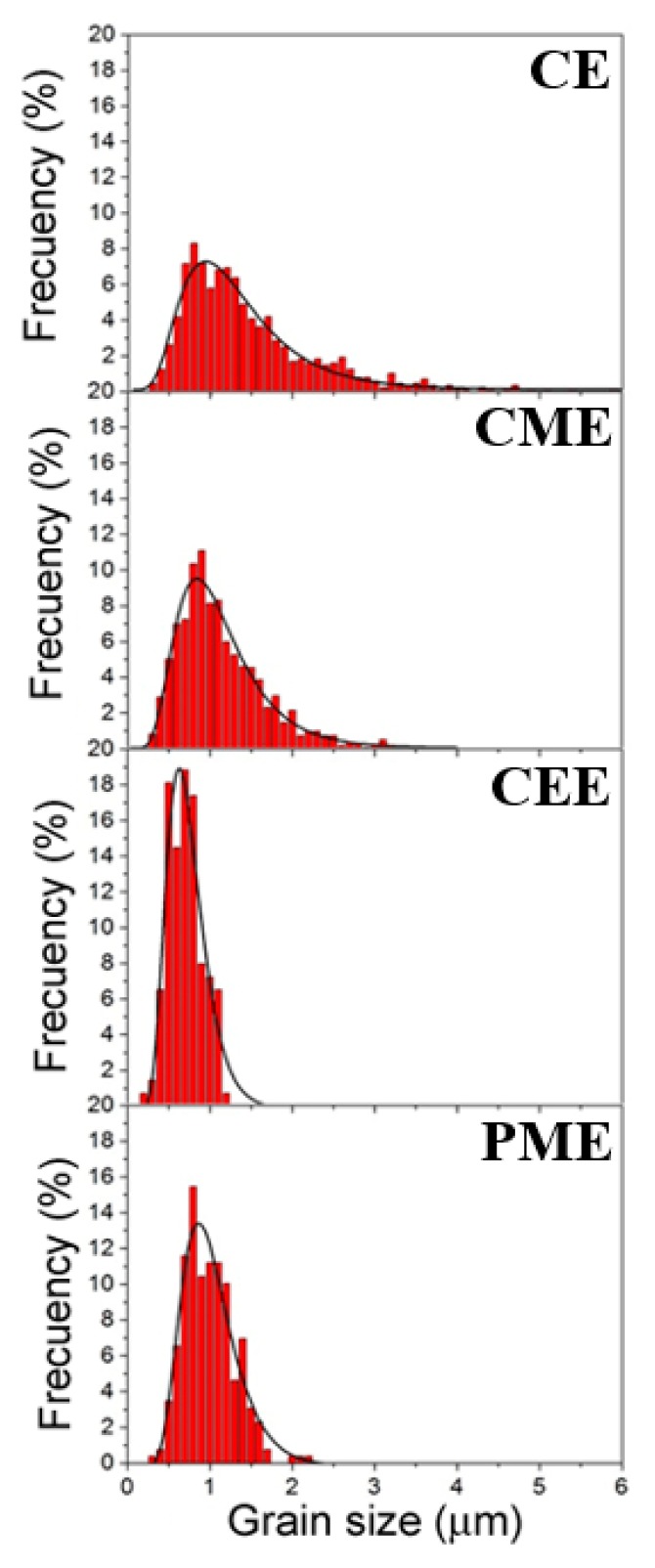
Grain histograms of processed Mg_98.5_Y_1_Zn_0.5_ alloy: CE, CME, CEE, and PME.

The grain size histogram of DRXed areas is showed in Figure 5. The CE material showed a bimodal grain structure with DRXed grains of about 1 µm and non-DRXed coarse grains elongated along the extrusion direction. It has been reported that DRXed grains are randomly oriented while coarse grains are oriented with the basal plane parallel to the extrusion direction [3]. The CME material was fully recrystallized with a grain size slightly lower than that of the extruded as-cast ingots (1.08 µm). The alloy processed using ECAP was not completely recrystallized. However, since both kinds of grains were intimately mixed, an accurate estimation of the volume fraction of both areas is not easily achieved without significant error. The DRXed grain size was lower than that for the CE material, 0.66 µm against 1.4 µm. Finally, the PME material was fully recrystallized with a grain size of 0.96 µm. The presence of the LPSO-phase as small particles rendered a more homogeneous distribution of this phase within the magnesium matrix, acting as pinning points which prevented further coarsening of the DRXed grains. This fact, however, did not lead to the alloy with the finest grain size, which was obtained in the alloy processed by ECAP, where the total processing strain was the highest (Table 1) and the processing temperature was the lowest.

**Figure 6:**
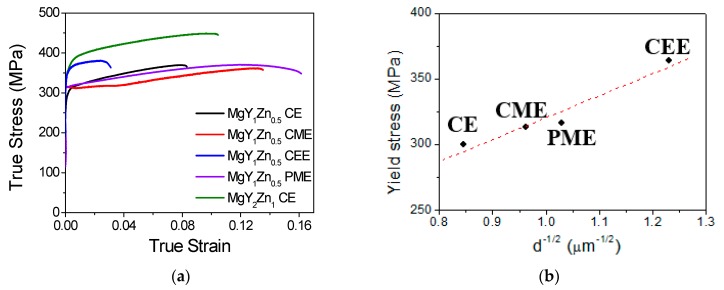
(**a**) True stress-true strain curves of processed Mg_98.5_Y_1_Zn_0.5_ alloy: CE, CME CEE, and PME. For comparison, the curve of the extruded Mg_97_Y_2_Zn_1_ alloy obtained from the as-cast alloy is included; (**b**) Evolution of the yield stress as a function of the inverse square root of the grain size for the alloy processed by the four routes: CE, CME, CEE, and PME.

Figure 6 shows the true stress-true strain curves of the Mg_98.5_Y_1_Zn_0.5_ alloy at room temperature for the four processing routes. For comparison, the tensile behavior of the extruded Mg_97_Y_2_Zn_1_ alloy is also shown [18]. It can be verified that lowering the yttrium and zinc content by the half, i.e., decreasing the volume fraction of the LPSO-phase in the alloys processed by the extrusion of as-cast ingots, induced a significant decrease of about 150 MPa, although the elongation to failure was enhanced.

The curves of materials processed by machined chips or RS powders, MCE and PME in Figure 6, revealed similar yield stresses, 313 and 316 MPa, respectively. These values are slightly higher than that of the conventional extruded material, 310 MPa, but the ultimate tensile stresses (UTS) are lower. Yamasaki et al. [3] proposed that coarse grains contribute to reinforce the alloy since neither basal slip nor tensile twinning can be easily activated in these grains. Garces et al. [19,20] confirmed this assumption using diffraction methods during in situ tension and compression tests. Moreover, they proved that the beginning of plasticity (yield stress) is always controlled by the activation of the basal slip system in DRXed grains. Therefore, the higher yield stress of CME and PME materials is due to their finer grain size. In the case of the CE alloy, coarse grains areas (non-DRXed grains) contribute to reinforce the material during tensile tests, reaching a yield stress almost comparable to that of CME and PME alloys. It is important to mention that the material produced using RS powders showed the higher elongation to failure. The use of the PM route in magnesium alloys produced extruded bars with low texture and, therefore, favored the ductility [21]. The Mg_98.5_Y_1_Zn_0.5_ processed by ECAP, the CEE material, attained the same yield stress as the Mg_97_Y_2_Zn_1_ alloy, although its ductility was highly reduced. This fact confirms again that the beginning of plasticity in this kind of alloys with bimodal grain structure is controlled by the plasticity of DRXed grains, so the grain refinement of these grains is the best strategy to improve the mechanical strength in these alloys. Figure 6b shows the variation of the yield stress with the reciprocal of the square root of the grain size (d^−1/2^). The yield stress increased as the grain size decreased following a Hall-Petch relationship with a slope of 170 MPa µm^−1/2^, similar to that obtained by Hagihara et al. [4] in Mg_97_Y_2_Zn_1_ alloy.

**Figure 7:**
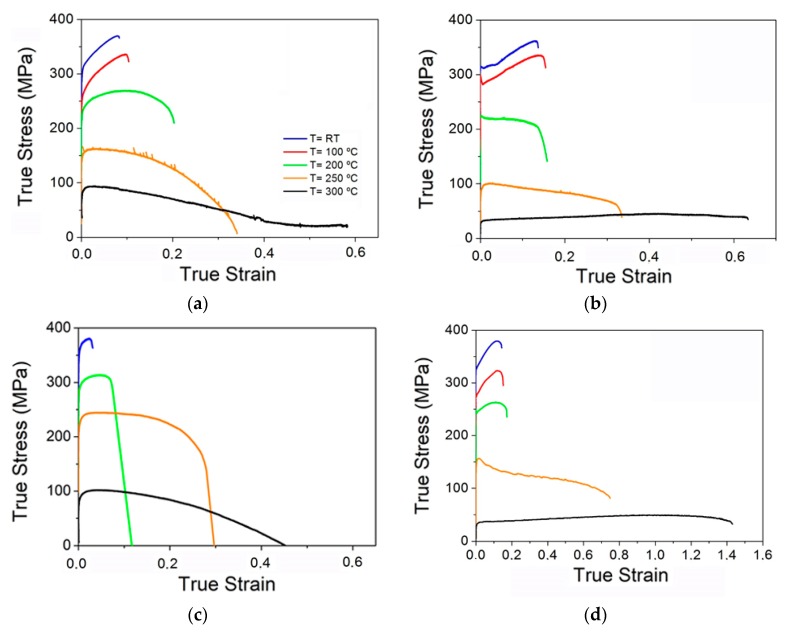
True stress-true strain curves in the temperature range of 25–300 °C. (**a**) CE, (**b**) CME, (**c**) CEE, and (**d**) PME.

**Figure 8:**
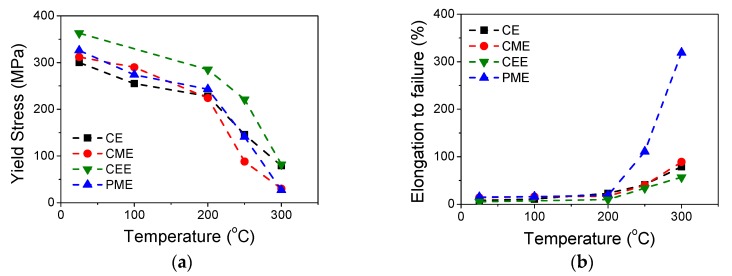
Evolution of the (**a**) yield stress and (**b**) elongation to failure as a function of the temperature for the four processing routes.

Figure 7 shows the true stress-true strain curves of the Mg_98.5_Y_1_Zn_0.5_ alloy for the four processing routes from RT to 300 °C at 10^−4^ s^−1^. For all processing routes, two ranges of behavior were distinguished: (1) the interval from RT up to 200 °C with high strength, high work hardening, and low ductility, and (2) the interval from 200 to 300 °C in which a pronounced decrease in strength was noticed but elongation to failure was substantially improved. The evolution of the yield stress is shown in Figure 8a from RT to 300 °C for the four processing routes. In all materials there was a gradual decrease in strength as the temperature increased, although the strength remained above 200 MPa up to 200 °C, and even up to 250 °C in the case of the CEE material. The alloy processed by extrusion followed by ECAP exhibited the highest yield stress over the entire temperature range. The mechanical behavior was determined mainly by two parameters: (i) the equiaxed or bimodal structure of the magnesium matrix, and (ii) the size and morphology of the LPSO-phase. The use of chips, powders resulted in fine-grained equiaxed magnesium grains. In addition, the alternative processing routes induced changes in the LPSO-phase. In the case of the CME material, the LPSO-phase was broken during the machining stage. In addition, the large strain accumulated during machining induced subsequent fracture during the extrusion process. In the CEE material, however, most of the LPSO-phase particles were not broken during ECAP. Instead, they were severely kinked. The distribution and alignment of the LPSO-phase was similar to that of the CE material, i.e., the LPSO-phase was arranged in long strings of particles aligned along the extrusion direction. However, the linearity of such arrangements was lower, especially in the case of the CEE material, compared to the CE alloy (as can be seen in Figure 1). A major difference of PME alloy was that the fine dendritic structure of the RS powders results in the fracture of LPSO-phase particles located at the interdendritic regions, leading to very homogenous distribution in the magnesium matrix. At room temperature and 100 °C, it seemed that the effect of grain size prevailed over the reinforcement due to the LPSO-phase. The reinforcing effect of LPSO-phase particles, larger than 0.5–1 µm in size, on the strength of magnesium alloys through the load transfer mechanism is well reported [20,22], due to its higher Young Modulus compared to the magnesium phase [22,23]. Thus, up to 100 °C, grain size is the main parameter controlling the yield stress of the different alloys. As the volume fraction of the LPSO-phase is essentially the same in all alloys and no large differences in the grain size of DRXed regions, changes in the reinforcing effect due to load transfer from the magnesium matrix to the LPSO-phase should be small, as a result of differences in the shape and size of the LPSO-phase particles.

Above 200 °C, both the specific characteristics of the magnesium matrix and the LPSO-phase particles in each alloy determined their mechanical behavior. Thus, the alloys with a fully recrystallized microstructure (CME and PME) became much softer than alloys with a bimodal microstructure (CE and CEE). Such different behavior was associated with some contribution of the grain boundary sliding (GBS) mechanism during the plastic deformation of the alloy. It is well reported that magnesium alloys containing the LPSO-phase exhibit superplasticity, even in alloys reinforced with volume fractions of coarse second phases exceeding 50% [6,24,25]. Consequently, GBS can proceed not only between magnesium grains but also in LPSO-phase/Mg interfaces. In alloys with grain sizes ranging between 2 and 5 µm and coarse second phases, superplastic behaviors with high elongations have been attained at temperatures above 300 °C because the accommodation of stresses generated during GBS must be achieved by cracking the LPSO-phase particles, which subsequently become homogeneously redistributed in the magnesium matrix. In addition, the existence of coarse non-DRXed regions precludes the activity of GBS there, reducing the superplastic deformation capabilities of the material. Superplastic behavior could take place once these coarse non-DRXed grains become recrystallized, usually at temperatures above 300 °C. Given the special features required for GBS in alloys containing the LPSO-phase, only the PME alloy exhibited superplastic behavior at 300 °C, 317% as elongation to failure (see Figure 8b), because this alloy was the only one combining the grain refinement of the magnesium matrix and the homogeneous distribution of fine second phases.

## 4. Conclusions

Severe deformation processing has been used to decrease the yttrium content of Mg-Y-Zn alloys containing LPSO phases without losing their mechanical strength at room temperature. In this study, three alternative routes were explored to maximize the mechanical strength of the Mg_98.5_Y_1_Zn_0.5_ alloy: the extrusion of machined chips and RS powders obtained from the cast alloy, and ECAP processing of the extruded bar. The microstructure of the extruded bars obtained from the machined chips and RS powders was fully recrystallized with a grain size near 1 µm, slightly improving the yield stress in comparison to the extruded alloy. On the other hand, the use of ECAP processing highly refined the grain size of the alloy up to 660 nm in such a way that its yield stress became similar to that of the extruded alloy with twice the yttrium content. Superplasticity could be achieved at 300 °C for the alloy prepared by a powder metallurgy route due to the grain size of the alloy, and the homogeneous distribution permitted the operation of grain boundary sliding.

## Figures and Tables

**Table 1:** Total processing strains, volume fraction of the LPSO-phase, non-recrystallized and recrystallized grains, recrystallized grain size, yield stress, σ_0.2_, ultimate tensile strength, Ultimate tensile strength (UTS), and tensile ductility for the processed Mg_98.5_Y_1_Zn_0.5_ alloy: CE, CME, CEE, and PME.

	ε_T_	f_LPSO_ (%)	f_Def_ (%)	f_DRX_ (%)	D (µm)	σ_0.2_ (MPa)	UTS (MPa)	Ductility (%)
CE	2.9	9 ± 0.7	45 ± 9	46 ± 8	1.4 ± 0.02	300	370	8
CME	3.6	9 ± 0.7	0	91	1.08 ± 0.02	313	361	13
CEE	4.2	9 ± 0.7	-	-	0.66 ± 0.02	364	383	3
PME	3.6	9 ± 0.7	0	91	0.96 ± 0.02	316	370	16
